# Measuring the influence of commercial determinants of health at the individual level: Protocol for tool development and validation

**DOI:** 10.1136/bmjopen-2026-118369

**Published:** 2026-08-19

**Authors:** Venkitachalam Ramanarayanan, Devika Krishna, Abhishek Mehta, Manu Raj Mathur

**Affiliations:** 1 Public Health Dentistry, Amrita Vishwa Vidyapeetham Amrita School of Dentistry, Kochi, Kerala, India; 2 Public Health Dentistry, Jamia Millia Islamia Faculty of Dentistry, New Delhi, New Delhi, India; 3 Centre for Dental Public Health and Primary Care, Queen Mary University of London Institute of Dentistry, London, UK

**Keywords:** Risk Factors, Surveys and Questionnaires, Delphi Technique, PUBLIC HEALTH

## Abstract

**Abstract:**

**Introduction:**

Existing frameworks on Commercial Determinants of Health (CDoH) largely focus on macro-level corporate practices and policy environments. Individual-level exposure—mediated through targeted marketing, pricing strategies, product availability and placement—remains underexplored, particularly in low- and middle-income countries. The absence of standardised tools to capture these exposures limits the ability to quantify commercial influences on health and weakens evidence-informed public health responses. There is a critical need for a robust, contextually grounded instrument to measure individual-level exposure to CDoH. Developing and validating an individual-level CDoH measurement tool is therefore essential to strengthen research, surveillance and policy action aimed at mitigating health-harming commercial influences. The availability of this tool will allow policymakers and public health practitioners to identify high-risk populations and monitor the effectiveness of regulatory actions addressing harmful commercial practices.

**Methods and analysis:**

This study will follow standard guidelines for tool (questionnaire) development. The process will include four sequential phases: item generation, refinement, validity testing and construct validation. A literature review with a systematic search strategy and in-depth interviews with purposively sampled adults will inform domain identification and item generation. An e-Delphi process involving experts in CDoH and public health will refine and confirm items followed by content validity. This will be followed by assessment of face validity for clarity and relevance. Construct validity will be assessed through exploratory and confirmatory factor analyses, using fit indices such as Minimum χ^2^/df, Goodness of Fit Index, Tucker-Lewis Index, Comparative Fit Index, Root Mean Square Error of Approximation and Standardised Root Mean Square Residual. Reliability will be estimated using Cronbach’s alpha, and convergent and discriminant validity will be examined through correlation analyses.

**Ethics and dissemination:**

Ethical approval has been obtained from the Institutional Ethics Committee, Amrita Institute of Medical Sciences, Kochi (ECASM-AIMS-2025-267). Results will be disseminated through peer-reviewed publications, conferences and policy briefs, and the validated tool will be made accessible for research and public health applications.

**STRENGTHS AND LIMITATIONS OF THIS STUDY**

- To the best of our knowledge, this protocol presents the first attempt at developing a validated tool to assess the influence of Commercial Determinants of Health on an individual level.
- Item generation will be done through literature review and integrated with qualitative interviews and psychometric testing will allow cross-verification of findings, reduce bias and build a robust conceptual foundation to the context.
- Standard process of questionnaire develepment - delphi consensus, validity, reliability, Exploratory Factor Analysis and Confirmatory Factor analysis will be done.
- The single-centre setting may limit external validity and generalisability to other regions and health system contexts.
- Self-reported data may be affected by recall and social desirability bias, influencing estimates of exposure to commercial influences.

## Introduction

 Commercial determinants of health (CDoH) are the ‘Strategies and approaches used by the private sector to promote products and choices that are detrimental to health’.^1^ It is also defined as ‘the private sector activities that have a direct or indirect, positive or negative impact on people’s health’.^2^ Thus, understanding the various private sector activities that affect public health, either favourably or unfavourably, as well as the underlying political, economic and normative frameworks as CDoH is crucial to identify the structures, products and operating policies that affect daily living conditions.^3^

The term CDoH was introduced by West and Marteau^4^ and popularised by Kickbusch *et al*.^1^ Initially focused on tobacco and alcohol, the concept has broadened to encompass ultra-processed foods, fossil fuels, gambling and automobiles. There is mounting evidence attributing modern epidemics of non-communicable diseases (NCDs) and health inequities to transnational corporations, with tobacco, ultra-processed foods, fossil fuels, and alcohol accounting for one-third of global deaths.^5^

CDoH scholarship predominantly addresses NCDs and has been focused on the food, tobacco and alcohol sectors in high-income areas.^6^ Current health frameworks and public health models generally focus on macro-level determinants, such as socioeconomic status, policy interventions, environmental influences and access to healthcare. Focus on influence of at the individual level are not sufficiently represented in current health frameworks.^7^ Individual-level influence of commercial determinants explain how personal behaviours are shaped by targeted marketing, pricing strategies or product availability are often not sufficiently represented. There is a gap in understanding how commercial factors directly affect an individual’s daily health choices. Frameworks might acknowledge the influence of the commercial sector (eg, the marketing of unhealthy foods), but do not adequately integrate or quantify how individuals internalise these influences at a personal level. Also, most studies on CDoH have been conducted in high-income countries, particularly those in North America and Western Europe.^8^ The reason could be better access to resources, established research infrastructure and a greater number of publicly available health-related datasets. But there is limited research exploring how commercial determinants affect individual’s health in low- and middle-income countries (LMICs). Therefore there is a need for a tool to evaluate how corporations affect health, along with the commercial determinants of global health in these countries.^9^

A validated CDoH assessment tool is essential to quantify commercial actors’ roles across disease spectra and populations, linking behavioural risk factors (eg, diet, tobacco use) to upstream determinants. This facilitates targeted mitigation of disparities, particularly among youth and socioeconomically disadvantaged groups, thereby advancing equitable public health strategies. This study aims to develop and validate a CDoH assessment tool focused on individual level determinants.

## Methods and analysis

**Figure 1:**
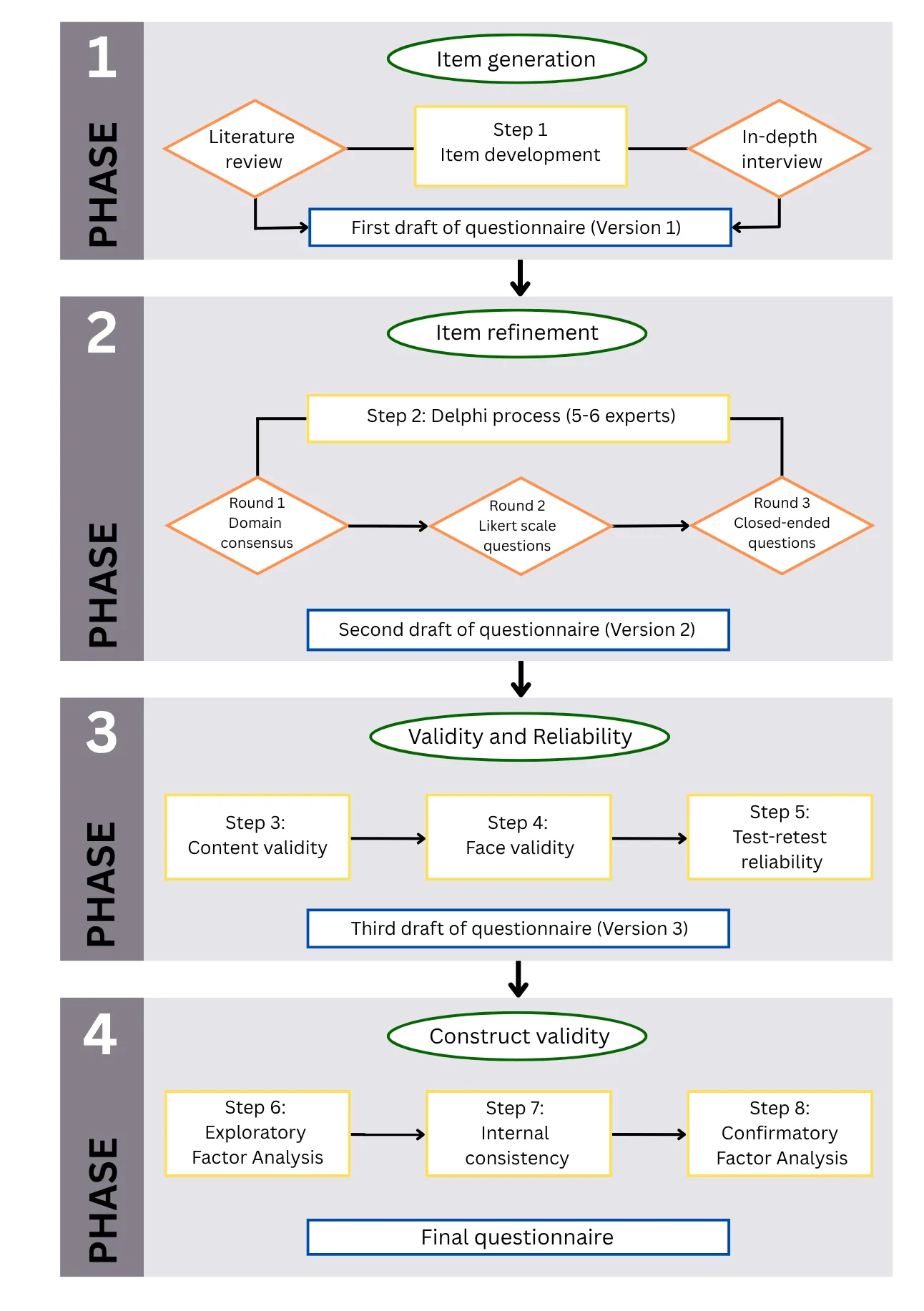
Proposed study flow diagram.

This protocol describes a questionnaire development process designed to develop and validate a tool to assess individual-level exposure to CDoH. The study will proceed in four phases: (1) item generation, (2) item refinement, (3) content validity and reliability testing followed by (4) construct validity (figure 1).

### Phase 1: Item generation

This phase will be accomplished in two stages. First, a literature review with a systematic search strategy will be conducted using databases PubMed and Scopus to map existing concepts, frameworks and instruments related to CDoH and individual-level exposure. The search strategy to be used for literature review is given in online supplemental file 1. This will inform the conceptual domains and guide item generation for the tool.^10^ In the second (qualitative) stage, in-depth interviews will be conducted with members of the general population using a semi-structured interview guide until data saturation is achieved. The interview guide will be prepared based on key domains in assessment of CDoH including marketing strategy, accessibility of products and product promotional activities. Participants will be purposively sampled to ensure diversity in age, gender, socioeconomic status and urban/rural residence. Thematic analysis of interview data, combined with insights from the literature review, will be used to generate an initial draft of domains and items (V1).

### Phase 2: Item refinement

Content refinement and item confirmation will be undertaken using an e-Delphi process (Step 2) with five to six experts in CDoH and areas of economics and public health. The experts identified include a mix of academicians in private and public sector, researchers in domain of public health, NCDs and economics working in government, private and non-profit think-tanks.

The e-Delphi method involves iterative rounds of expert questionnaires to achieve consensus on complex topics. Its methodological rigour ensures reliable, transparent results through structured steps.^11^ Key elements include identifying the research problem, constructing the initial questionnaire via literature review (including grey literature), selecting a diverse expert panel, distributing questionnaires with controlled feedback, analysing data for consensus and reporting findings.^11^ Rigour demands predefining consensus (eg, 70% agreement or statistical measures like Cronbach’s alpha), documenting expert selection/exclusion, providing anonymised feedback after each round to minimise bias and justifying termination criteria.^12^ For the same, we would be using Welphi, a specialised online platform for implementing e-Delphi studies, automating iterative rounds with anonymous feedback.^13^ At the end of this phase, the second draft of the questionnaire (V2) is expected to be ready.

### Phase 3: Validity and Reliability

Assessing the validity of the questionnaire will be undertaken by the following steps:

Step 3: Assessment of content validity: Assessment of Content Validity Index (for both item and scale) will be done with another set of five experts identified from literature working on the domain of CDoH, health economists and researchers in the domain of public health and NCDs using the Content Validity Index.^14^

Step 4: Assessment of face validity: The face validity will be assessed by the clarity and comprehensibility of all items in each domain through the respondents’ rating. Respondents will be asked to rate the clarity and comprehensibility for all items in each domain according to a four-interval scale (1 = not clear and understandable; 2 = somewhat clear and understandable; 3 = clear and understandable and 4 = very clear and understandable). Ratings 1 and 2 represent invalid or irrelevant content, while ratings 3 and 4 represent valid or relevant content based on its clarity and comprehension. Item-level Face validity Index (FVI) and scale-level FVI will be calculated. Values can range from 0 to 1.^15^ The calculated FVI of at least 0.80 will be taken as the acceptable FVI value.^16^

Step 5: Test–retest reliability: Following face validity, the questionnaire will be subjected to test–retest reliability by distributing it twice with a gap of 2 weeks to a group of 30 participants recruited from the general public. This will measure the degree of consistency exhibited when a measurement is repeated under identical conditions. As the responses are measured on an ordinal scale, weighted kappa will be used to assess the reliability of each item. Intraclass correlation coefficient for domain and scale will be calculated. At the end of this stage, the third draft of the questionnaire is expected to be complete (V3).

### Phase 4: Construct validity

As the questionnaire is expected to have domains, a construct validity will be performed. It is the degree to which an instrument indeed measures the latent dimension or construct for which it was developed to evaluate (also known as scale validity). This will be done among a sample recruited from the general public (end-users). The draft scale will be administered to a larger sample drawn from adults (≥18 years) from the general public, including patients and bystanders attending the outpatient departments of Amrita School of Dentistry and its affiliated hospitals. Individuals unwilling to participate and those employed in commercial sectors directly linked to health-harming products (eg, tobacco, processed foods, sugar-sweetened beverages) will be excluded. Sampling will be consecutive and convenient, with efforts to ensure representation across age, gender and socioeconomic strata.^17^

Step 6: Exploratory factor analysis

Construct validity of the questionnaire will be initially evaluated using Exploratory Factor Analysis (EFA) to identify the underlying factor structure of the instrument. Prior to EFA, the suitability of the data for factor analysis will be assessed using the Kaiser-Meyer-Olkin measure of sampling adequacy and Bartlett’s test of sphericity. Factors will be extracted using Principal Axis Factoring with Direct Oblimin rotation, considering the anticipated correlation between factors. The number of factors retained will be determined based on eigenvalues greater than 1, examination of the scree plot and conceptual interpretability of the factor structure. Items with low factor loadings (<0.40), substantial cross-loadings or poor conceptual fit will be considered for removal or reassignment, and EFA will be repeated iteratively until a stable and interpretable factor structure is achieved.

Step 7: nternal consistency

Following finalisation of the factor structure, the internal consistency reliability of each domain and the overall questionnaire will be assessed using Cronbach’s alpha coefficient. Corrected item–total correlations and Cronbach’s alpha if item deleted will be examined to evaluate the contribution of individual items to their respective domains. Floor and ceiling effects will also be assessed by determining the proportion of respondents achieving the minimum and maximum possible scores for each domain and the overall questionnaire, with proportions exceeding 15% indicating the presence of floor or ceiling effects.

Step 8: Confirmatory Factor Analysis

The final draft of the questionnaire will subsequently undergo Confirmatory Factor Analysis (CFA) using an independent sample to validate the factor structure identified through EFA. Model fit will be evaluated using multiple fit indices, including the χ^2^/df ratio, Comparative Fit Index, Tucker-Lewis Index, Root Mean Square Error of Approximation and Standardised Root Mean Square Residual. Convergent validity will be assessed using standardised factor loadings, composite reliability and average variance extracted, while discriminant validity will be evaluated using established criteria to confirm the distinctiveness of the identified constructs.

#### Sample size

In-depth interviews will be conducted until data saturation is achieved. The Delphi process will involve seven to eight domain experts to ensure content relevance and expert consensus.^18^ Content validity of the developed questionnaire will be done with another group of five experts.^15^ Face validity of the instrument will be assessed through a pilot study conducted among 20 participants. Test–retest reliability will be assessed in a group of 30 participants. For EFA, the sample size will be determined based on the conventional item-to-respondent ratio of 1:10, with 10 participants per scale item as per the rule of thumb.^19^ For CFA, the third draft of the questionnaire will be tested among 200 participants.

#### Expected outcomes

The primary outcome is a newly developed scale to influence the effect of CDoH on an individual. The tool could help health professionals design targeted interventions for individuals. For instance, it might reveal patterns of unhealthy eating habits linked to exposure to specific forms of advertising which could then prompt an intervention focusing on healthy beverage choices.

It could also function as a means to track changes in exposure and behaviour over time. For instance, after an individual engages in an intervention aimed at reducing exposure to unhealthy advertisements, the tool could be used to assess whether there are any changes in the individual’s health behaviours. Health educators could use the tool to teach individuals how to identify and resist commercial influences, particularly when it comes to food, beverages and lifestyle choices. Health educators could use the tool to teach individuals how to identify and resist commercial influences, particularly when it comes to food, beverages and lifestyle choices.

Another key expected outcome of this tool tailored for LMICs is to enable systematic and comparable assessment of individual-level exposure to commercial influences on health, addressing a critical measurement gap in LMIC settings. Its use in future research is expected to generate high-quality empirical evidence to inform policy, guide interventions and strengthen evidence-informed public health decision-making.

### Ethics and dissemination

The ethical approval for the study has been obtained from the Institutional Ethics Committee of Amrita Institute of Medical Sciences, Kochi, Kerala, India (ECASM-AIMS-2025-267). Written/online informed consent will be obtained from all participants after explaining the study purpose, procedures, risks and benefits in the local language (Malayalam/English). Participation will be voluntary, and participants will be informed that they can withdraw at any time without any disadvantage. All data will be anonymised and used solely for research purposes.

Privacy and confidentiality will be ensured by assigning unique study IDs instead of names, storing electronic data on password-protected, encrypted devices with restricted access, limiting data access to the research team and supervisors only, and securely destroying paper records after data entry and archiving. Potential risks (eg, discomfort when discussing commercial practices, concerns about privacy) will be minimised through interviewer training in sensitive communication, conducting interviews in private settings and providing information on available support services. No direct compensation will be provided.

Findings will be disseminated through peer-reviewed publications in public health and health policy journals, presentations at national and international conferences. The final validated CDoH exposure tool will be made available for use in research with detailed documentation on its development and recommended use.^20^

## Supplementary material

10.1136/bmjopen-2026-118369 online supplemental file 1
