# Supplementary material for: Measuring the influence of commercial determinants of health at the individual level: Protocol for tool development and validation
Source: BMJ Open. 2026 Aug 19;16(8):e118369. doi: 10.1136/bmjopen-2026-118369 (PMC13504878; doi:10.1136/bmjopen-2026-118369)
Supplement: Supplementary file 1 [file bmjopen-2026-118369.reviewer_comments.pdf]

PEER REVIEW HISTORY

BMJ Open publishes all reviews undertaken for accepted manuscripts. Reviewers are asked to complete a checklist review form and are provided with free text boxes to elaborate on their assessment. These free text comments are reproduced below.

ARTICLE DETAILS

Title (Provisional)

Measuring the Influence of Commercial Determinants of Health at the Individual Level: Protocol for Tool Development and Validation

Authors

Ramanarayanan, Venkitachalam; Krishna, Devika; mehta, abhishek; Mathur, Manu Raj

---

VERSION 1 - REVIEW

**Reviewer**	**1**
**Name**	**Aoyama, Maho**
**Affiliation**	**Tohoku University Graduate School of Medicine**
**Date**	**28-Apr-2026**
**COI**	**None**

I appreciate the opportunity to review this manuscript.

This is an interesting protocol manuscript addressing an important and relevant topic. The proposed study is meaningful, and the process of scale development is generally consistent with established methodological approaches.

However, if the authors intend to position this study within a mixed methods framework, further clarification is needed. In mixed methods research, the key issue is not merely the use of both qualitative and quantitative methods, but the extent to which these components are theoretically and methodologically integrated.

To strengthen the manuscript and ensure appropriate alignment with mixed methods methodology, I would recommend the following revisions:

#1 Clarification of study design

Please state that the study adopts an exploratory sequential mixed methods design, in which qualitative findings are used to inform the development of quantitative measures.

#2 Explicit description of integration

The manuscript should clearly describe how integration is achieved. It would be important to go beyond stating that both qualitative and quantitative methods are used, and instead articulate how the qualitative findings are connected to item generation, and how the results from both phases will be interpreted in relation to each other.

In addition, if the authors do not intend to further elaborate on integration, it may be more appropriate to frame the study as a conventional scale development protocol rather than as mixed methods research.

Clarifying these points will improve the methodological transparency and strengthen the justification for framing the study as mixed methods research.

VERSION 1 - AUTHOR RESPONSE

Reviewer: 1
Dr. Maho Aoyama, Tohoku University Graduate School of Medicine, Yamagata Prefectural University of Health Sciences
Comments to the Author:
I appreciate the opportunity to review this manuscript.
This is an interesting protocol manuscript addressing an important and relevant topic. The proposed study is meaningful, and the process of scale development is generally consistent with established methodological approaches.

Response: Thank you for your words of appreciation

However, if the authors intend to position this study within a mixed methods framework, further clarification is needed. In mixed methods research, the key issue is not merely the use of both qualitative and quantitative methods, but the extent to which these components are theoretically and methodologically integrated.

To strengthen the manuscript and ensure appropriate alignment with mixed methods methodology, I would recommend the following revisions:

#1 Clarification of study design
Please state that the study adopts an exploratory sequential mixed methods design, in which qualitative findings are used to inform the development of quantitative measures.

Response: We thank you for the above comments. This study was not intended to integrate the qualitative and quantitative findings rather qualitative data was collected in one step to inform the item generation. We understand that this is not ideal to be called Mixed-methods research and hence have changed it to tool (questionnaire) development.

#2 Explicit description of integration
The manuscript should clearly describe how integration is achieved. It would be important to go beyond stating that both qualitative and quantitative methods are used, and instead articulate how the qualitative findings are connected to item generation, and how the results from both phases will be interpreted in relation to each other.

In addition, if the authors do not intend to further elaborate on integration, it may be more appropriate to frame the study as a conventional scale development protocol rather than as mixed methods research.
Response: We have made the changes as suggested.

Clarifying these points will improve the methodological transparency and strengthen the justification for framing the study as mixed methods research.

VERSION 2 - REVIEW

**Reviewer**	**1**
**Name**	**Aoyama, Maho**
**Affiliation**	**Tohoku University Graduate School of Medicine**
**Date**	**01-Jul-2026**
**COI**	

Thank you for your revision. I do not have further comments.

VERSION 2 - AUTHOR RESPONSE

Reviewer: 1
Dr. Maho Aoyama, Tohoku University Graduate School of Medicine, Yamagata Prefectural University of Health Sciences
Comments to the Author:
Thank you for your revision. I do not have further comments.

Response: Thank you for considering our revised version.
