# Supplementary material for: Measuring the influence of commercial determinants of health at the individual level: Protocol for tool development and validation
Source: BMJ Open. 2026 Aug 19;16(8):e118369. doi: 10.1136/bmjopen-2026-118369 (PMC13504878; doi:10.1136/bmjopen-2026-118369)
Supplement: Supplementary file 2 [file bmjopen-16-8-s001.docx]

**Search Strategy for Phase 1 (Step 1) – Item generation**

**PUBMED**

((("commercial determinants of health"[tiab] OR "corporate determinants of health"[tiab])) AND ((consumer*[tiab] OR individual*[tiab] OR public[tiab] OR "purchasing behaviour"[tiab] OR "consumer behavior"[Mesh] OR "health behavior"[MeSH]))) AND (("Marketing"[Mesh] OR "Advertising"[Mesh] OR marketing[tiab] OR advertising[tiab] OR "digital marketing"[tiab] OR "social media marketing"[tiab] OR sponsorship[tiab] OR promotion[tiab] OR branding[tiab] OR "corporate social responsibility"[tiab] OR CSR[tiab] OR price*[tiab] OR discount*[tiab] OR affordability[tiab] OR availability[tiab] OR "point of sale"[tiab]))

**SCOPUS**

( TITLE-ABS ( "commercial determinants of health" ) OR TITLE-ABS ( "corporate determinants of health" ) ) AND ( TITLE-ABS ( consumer* ) OR TITLE-ABS ( individual* ) OR TITLE-ABS ( public ) OR TITLE-ABS ( "purchasing behaviour" ) OR INDEXTERMS ( "consumer behavior" ) OR INDEXTERMS ( "health behavior" ) ) AND ( INDEXTERMS ( Marketing ) OR INDEXTERMS ( Advertising ) OR TITLE-ABS ( Marketing ) OR TITLE-ABS ( Advertising ) OR TITLE-ABS ( "digital marketing" ) OR TITLE-ABS ( "social media marketing" ) OR TITLE-ABS ( sponsorship ) OR TITLE-ABS ( promotion ) OR TITLE-ABS ( branding ) OR TITLE-ABS ( "corporate social responsibility" ) OR TITLE-ABS ( CSR ) OR TITLE-ABS ( price* ) OR TITLE-ABS ( discount* ) OR TITLE-ABS ( affordability ) OR TITLE-ABS ( availability ) OR TITLE-ABS ( "point of sale" ) )
